# BADGE: A novel Bayesian model for accurate abundance quantification and differential analysis of RNA-Seq data

**DOI:** 10.1186/1471-2105-15-S9-S6

**Published:** 2014-09-10

**Authors:** Jinghua Gu, Xiao Wang, Leena Halakivi-Clarke, Robert Clarke, Jianhua Xuan

**Affiliations:** 1 Department of Electrical and Computer Engineering, Virginia Polytechnic Institute and State University, Virginia, USA; 2 Department of Oncology, Lombardi Comprehensive Cancer Center, Georgetown University, Washington, DC, USA

## Abstract

**Background:**

Recent advances in RNA sequencing (RNA-Seq) technology have offered unprecedented scope and resolution for transcriptome analysis. However, precise quantification of mRNA abundance and identification of differentially expressed genes are complicated due to biological and technical variations in RNA-Seq data.

**Results:**

We systematically study the variation in count data and dissect the sources of variation into between-sample variation and within-sample variation. A novel Bayesian framework is developed for joint estimate of gene level mRNA abundance and differential state, which models the intrinsic variability in RNA-Seq to improve the estimation. Specifically, a Poisson-Lognormal model is incorporated into the Bayesian framework to model within-sample variation; a Gamma-Gamma model is then used to model between-sample variation, which accounts for over-dispersion of read counts among multiple samples. Simulation studies, where sequencing counts are synthesized based on parameters learned from real datasets, have demonstrated the advantage of the proposed method in both quantification of mRNA abundance and identification of differentially expressed genes. Moreover, performance comparison on data from the Sequencing Quality Control (SEQC) Project with ERCC spike-in controls has shown that the proposed method outperforms existing RNA-Seq methods in differential analysis. Application on breast cancer dataset has further illustrated that the proposed Bayesian model can 'blindly' estimate sources of variation caused by sequencing biases.

**Conclusions:**

We have developed a novel Bayesian hierarchical approach to investigate within-sample and between-sample variations in RNA-Seq data. Simulation and real data applications have validated desirable performance of the proposed method. The software package is available at http://www.cbil.ece.vt.edu/software.htm.

## Background

Next Generation Sequencing (NGS) technology has opened a new era for transcriptome analysis, which grants the ability to investigate novel biological problems, such as alternative splicing, differential isoforms, gene fusion, etc. By piling up millions of reads along the reference genome, RNA-Seq technology can obtain signals in a much larger dynamic range with much higher accuracy compared to traditional microarray based technologies. For RNA-Seq analysis, the most popular routine is to determine the expression of genes (abundance quantification) and to identify differentially expressed genes (DEGs).

Methods that quantify gene/isoform level expression mainly fall into two categories: Poisson count mode (e.g., Cufflinks [1], etc) and linear regression model (e.g., Isolasso [2], SLIDE [3], BASIC [4], etc.). The major challenge in accurate quantification of gene expression is that large systematic bias in sequencing counts has been observed due to multiple factors. In contrast to uniform assumption of read distribution, it has been reported that sequence counts show a variety of physical and chemical biases, including transcript length bias, GC-content bias, random hexamer priming bias, etc [5-7].

Differential analysis of RNA-Seq data has been focused on modeling variance among biological replicates or samples in the same phenotype group. EdgeR [8] is the first method that models the 'between-sample' variability by replacing the Poisson model with Negative Binomial model. Various statistical methods have been proposed to model the variance among samples in biological groups, aimed to improve overall fitting of count data or robustness against outliers [9-12].

Despite initial success to model uncertainties associated with sequencing counts from different aspects, there lacks a systematic effort to address variability in RNA-Seq data. We dissect the variance in sequencing counts along two dimensions: over-dispersion of read counts within the same sample (i.e., within-sample variation) and over-dispersion of read counts among individuals from the same biological group (i.e., between-sample variation). Within-sample variation typically leads to large variance of read counts among genomic loci (e.g., nucleotides or exons), which have similar expression level in the same sample. It is typically caused by technical artifacts such as uncorrected systematic bias and gene specific random effects. On the other hand, between-sample variation is mostly due to biological differences among samples under the same condition. To increase the accuracy of abundance estimation so as to improve DEG identification, immediate attention is needed to developing a unified model that takes care of both forms of variation in RNA-Seq data. We propose a computational method, namely Bayesian Analysis of Dispersed Gene Expression ('BADGE'), to model variability in RNA-Seq data. A full Bayesian model is employed to simultaneously account for within-sample variation and between-sample variation to improve inference. The proposed method has several novel contributions compared to existing methodologies: 1) a unified Bayesian causality model is developed for joint abundance estimation and DEG identification. The improved accuracy in profiling mRNA abundance can facilitate the identification of DEGs, which may in turn refine the parameter learning in abundance quantification. 2) A Poisson-Lognormal regression model is incorporated to model within-sample variation [13]. Instead of dealing with multiples sources of technical bias and variation separately, the proposed method can 'blindly' detect over-dispersion pattern within the individual sample. 3) Gamma-Gamma model [14] is used to model between-sample variation, which accounts for over-dispersion of read counts among multiple samples. BADGE is a unified computational method that extensively models variability in RNA-seq data to improve abundance quantification and DEG identification.

## Methods

### Bayesian Analysis of Dispersed Gene Expression (BADGE)

We have developed a computational method, namely Bayesian Analysis of Dispersed Gene Expression (BADGE), to model extensive variability in RNA-Seq data. BADGE explicitly models both between-sample variation and within-sample variation to improve abundance quantification and DEG identification. In this paper, we only focus on the gene level analysis, while the concept can be straight-forwardly generalized for genes with multiple transcripts (isoforms).

Let *y*_*g,i,j *_represent observed counts that fall into the *i*^th ^(1 ≤ *i *≤ *I*_g_) exon region of gene *g *(1 ≤ *g *≤ *G*) in sample *j *(1 ≤ *j *≤ *J*), which follows Poisson distribution with mean *γ*_*g,i,j*_. *I*_*g *_is the number of exons in gene *g. G *is the total number of genes. *J *= *J*_1 _+ *J*_2 _is the total number of samples, where *J*_1 _and *J*_2 _denote samples in condition 1 and 2, respectively. Within-sample over-dispersion indicates that *γ*_*g,i,j *_has unknown heterogeneity across the gene rather than taking constant value. A hierarchical Bayesian model is constructed to model within-sample variation of RNA-Seq data as follows:

(1) $$y_{g,i,j}\sim Poiss\left(\gamma_{g,i,j}\right),$$

(2) $$\gamma_{g,i,j}=x_{g,i}\beta_{g,j}\text{exp}\left(U_{g,i,j}\right),$$

(3) $$U_{g,i,j}\sim N\left(0,\tau \right),\;\text{s}\text{.t}\text{.}\;\underset{i}{\sum }U_{g,i,j}=0,$$

(4) τ~Gamma(a,b),

where *β*_*g,j *_is the true expression level of gene *g *for sample *j. x*_*g,i *_is the length of the *i*^th ^exon weighted by the library size of sample *j. U*_*g,i,j *_is the unknown within-sample variation parameter, which follows normal distribution with mean 0 and precision *τ*. 'Flat' prior is assigned for *τ *by setting its shape *a *= 1 and rate *b *= 0. Equations (1-4) are also known as the Poisson-Lognormal regression model with identity link function.

**Figure 1:**
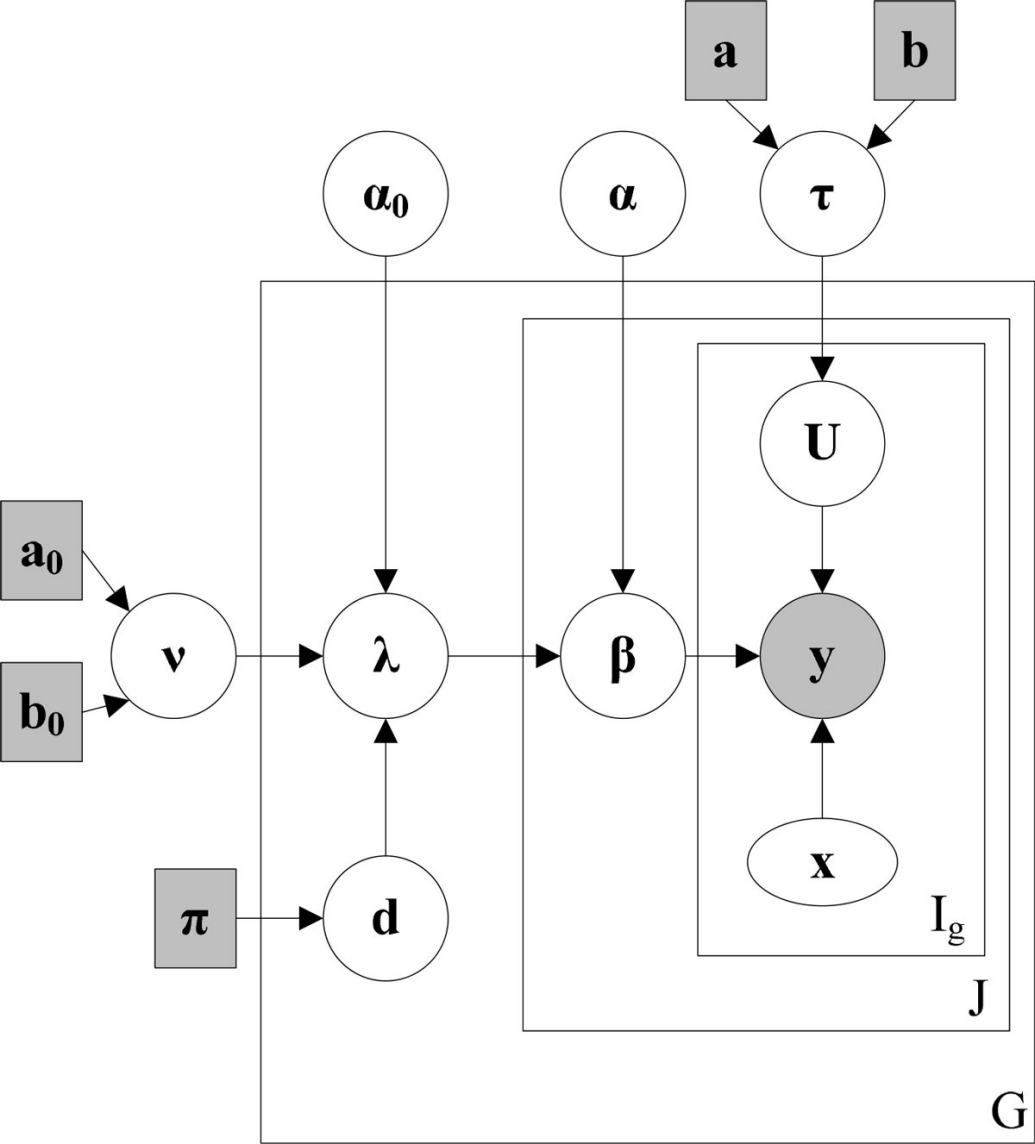
**Dependency graph of model parameters in BADGE**.Observation (read count) **y **is shaded, while the random variables are denoted as circles. Fixed parameters are denoted as shaded squares. Exon length information **x **is denoted as oval. *I_g _*is the number of exons, *J *is the total number of samples in two groups, and *G *is the number of genes. Note that parameter **λ **actually is a parameter set {**λ**^(1)^, **λ**^(2)^}, which correspond to two sample conditions.

Not only does the read count *y*_*g,i,j *_exhibits over-dispersion, but also *β*_*g,j *_has variation across multiple samples in the same biological group. To model between-sample variation carried by *β*_*g,j*_, we adopt the Gamma-Gamma model that is widely used in microarray gene expression analysis [14] into the Poisson count model. Let *j*_1 _and *j*_2 _represent samples in condition 1 and 2. *d_g _*is the binary differential state of gene *g*, where *d_g _*= 0 means gene *g *is not differentially expressed; *d_g _*= 1, otherwise. The Gamma-Gamma model for RNA-Seq differential expression is given by:

(5) $$\text{If}\;d_{g}=0,\;\beta_{g,j}\sim Gamma\left(\alpha ,\lambda_{g}\right),$$

(6) $$\lambda_{g}\sim Gamma\left(\alpha_{0},v\right),$$

(7) $$\text{or}\;\text{if}\;dg=1,\;\beta_{g,j_{1}}\sim Gamma\left(\alpha ,\lambda_{{}_{g}}^{\left(1\right)}\right),\beta_{g,j_{1}}\sim Gamma\left(\alpha ,\lambda_{{}_{g}}^{\left(2\right)}\right)$$

(8) $$\lambda_{g}^{\left(1\right)},\lambda_{g}^{\left(2\right)}\sim Gamma\left(\alpha_{0},v\right),$$

(9) $$\text{and}\;v\sim Gamma\left(a_{0},b_{0}\right),$$

where $\lambda_{g}^{\left(1\right)}$ and $\lambda_{g}^{\left(2\right)}$ are the rate parameters of Gamma distribution. If *d_g _*= 0, $\lambda_{g}^{\left(1\right)}=\lambda_{g}^{\left(2\right)}=\lambda_{g}$; if $d_{g}=1,\lambda_{g}^{\left(1\right)}\neq \lambda_{g}^{\left(2\right)}$. *α *is the shape parameter for *β*_*g,j*_, which does not depend on differential state *d_g _*Moreover, we assume that the pooled rate parameter *λ_g _*from the gene population further follows Gamma distribution with shape parameter *α_0 _*and rate parameter *v*. We assign non-informative priors for hyper-parameters *α, α_0_, d_g_*(*P*(*d_g _*= 1)= *π_g _*= 0.5) and *v*(*a_0 _*= 1, *b_0 _*= 0). The sub-model defined by Equations (5-9) considers between-sample variation within the same group, which borrows knowledge from the entire population to improve parameter estimation of individual genes. Figure 1 gives the Bayesian hierarchical dependency graph for all the parameters involved in the BADGE method using plate notation. There are three plates in Figure 1: The inner plate denotes dependency among read counts within *I_g _*exons in gene *g*; middle plate denotes dependency among *J *samples; and the outmost plate represents *G *genes. Observation **y**, represented by shaded circle, is the raw exon level RNA-Seq count (for gene level count data, *I_g _*= 1), which depends on mRNA abundance **β**, gene design matrix **x **and within-sample over-dispersion parameter **U**. **β **further depends on group level between-sample over-dispersion Gamma parameter **λ **and *α*, and **U **depends on global within-sample over-dispersion parameter *τ*. Parameter **λ **is determined by gene level differential state **d**, and its priors *ν *and *α_0_. d_g _*(differential state of gene *g*) is assumed to follow P(dg = 1) = *π_g _*= 0.5. Hyper-parameters *a*_0_, *b_0_, π, a*, and *b*, shown in shaded square, are fixed to construct non-informative priors (see additional file 1).

### Estimate model parameters using Gibbs sampling

The joint posterior distribution of all parameters given observation **y **(read count) and **x **(exon length) is given by:

(10) $$\begin{array}{c} P\left(\beta ,U,\tau ,\lambda ,d,\alpha ,\alpha_{0},v|y,x\right)\\ \sim P\left(y|\beta ,U,\tau ,\lambda ,d,\alpha ,\alpha_{0},v,x\right)P\left(\beta ,U,\tau ,\lambda ,d,\alpha ,\alpha_{0},v|x\right)\\ \sim P\left(y|\beta ,x,U,\tau ,\right)P\left(U|\tau \right)P\left(\beta |\lambda ,\alpha \right)P\left(\lambda |d,\alpha_{0},v\right)P\left(\tau \right)P\left(d\right)P\left(\alpha \right)P\left(\alpha_{0}\right)P\left(v\right)\\ \sim \underset{g}{\prod }\underset{j_{1}}{\prod }\underset{i}{\prod }\left(\frac{{\left(x_{g,i}\beta_{{}_{g,j_{1}}}\text{exp}\left(U_{g,i,j_{1}}\right)\right)}^{y_{g,i,j_{1}}}}{y_{g,i,j_{1}}!}.e^{-x_{g,i}\beta_{g,j1}\text{exp}\left(U_{g,i,j_{1}}\right)}\right)\\ \times \underset{g}{\prod }\underset{j_{2}}{\prod }\underset{i}{\prod }\left(\frac{{\left(x_{g,i}\beta_{{}_{g,j_{2}}}\text{exp}\left(U_{g,i,j_{2}}\right)\right)}^{y_{g,i,j_{2}}}}{y_{g,i,j_{2}}!}.e^{-x_{g,i}\beta_{g,j_{2}}\text{exp}\left(U_{g,i,j_{2}}\right)}\right)\\ \times \underset{g}{\prod }\underset{j}{\prod }\underset{i}{\prod }{\sqrt{\tau e}}^{-\tau \frac{U_{g,i,j}^{2}}{2}}\\ \times \underset{g}{\prod }\left(\underset{j_{1}}{\prod }\frac{{\left(\lambda_{g}^{\left(1\right)}\right)}^{\alpha }}{\text{Γ}\left(\alpha \right)}\beta_{g,j_{1}}^{\alpha -1}e^{-\lambda_{g}^{\left(1\right)}\beta_{g,j_{1}}}\times \underset{j_{2}}{\prod }\frac{{\left(\lambda_{g}^{\left(2\right)}\right)}^{\alpha }}{\text{Γ}\left(\alpha \right)}\beta_{g,j_{2}}^{\alpha -1}e^{-\lambda_{g}^{\left(2\right)}\beta_{g,j_{{}_{2}}}}\right)\\ \times \underset{g}{\prod }{\left(\frac{v^{\alpha_{0}}}{\text{Γ}\left(\alpha_{0}\right)}{\left(\lambda_{g}^{\left(1\right)}\right)}^{\alpha_{0}-1}e^{-v\lambda_{g}^{\left(1\right)}}\times \frac{v^{\alpha_{0}}}{\text{Γ}\left(\alpha_{0}\right)}{\left(\lambda_{g}^{\left(2\right)}\right)}^{\alpha_{0}-1}e^{-v\lambda_{g}^{\left(2\right)}}\right)}^{d_{g}}\\ \times \underset{g}{\prod }{\left[\frac{v^{\alpha_{0}}}{\text{Γ}\left(\alpha_{0}\right)}{\left(\lambda_{g}^{\left(1\right)}\right)}^{\alpha_{0}-1}e^{-v\lambda_{g}^{\left(1\right)}}\times \text{I}\left(\lambda_{g}^{\left(1\right)}-\lambda_{g}^{\left(2\right)}\right)\right]}^{1-d_{g}}\\ \times \frac{b^{a}}{\text{Γ}\left(a\right)}\tau^{a-1}e^{-b\tau }\times \underset{g}{\prod }\pi_{g}\times \frac{b_{0}^{a_{0}}}{\text{Γ}\left(a_{0}\right)}v^{a_{0}-1}e^{-b_{0}v}, \end{array}$$

For Poisson-Lognormal regression model, the posterior distributions of parameters *β_g,j_, U_g,i,j _*and τ can be sampled from their corresponding conditional distributions as:

(11) $$\begin{array}{c} P\left(\beta_{g,j}|y,U_{g}\right)\sim \beta_{g,j}^{\underset{i}{\sum }y_{g,i,j}}\left(-\beta_{g,j}\underset{i}{\sum }x_{g,i}\text{exp}\left(U_{g,i,j}\right)\right)\\ \;\;\;\;\;\;\;\;\sim Gamma\left(\underset{i}{\sum }y_{g,i,j}+1,\underset{i}{\sum }x_{g,i}\text{exp}\left(U_{g,i,j}\right)\right), \end{array}$$

(12) $$P\left(U_{g,i,j}|\beta_{g,j},\tau ,y\right)\sim \text{exp}{\left(U_{g,i,j}\right)}^{y_{g,i,j}}\times \text{exp}\left(-\beta_{g,j}x_{g,i}\text{exp}\left(U_{g,i,j}\right)\right)\times \text{exp}\left(-\frac{\tau U_{g,i,j}^{2}}{2}\right),$$

(13) $$\begin{array}{c} P\left(\tau |U\right)\sim \tau^{a-1+\frac{J\underset{g}{\sum }I_{g}}{2}}\times \text{exp}\left(-\left(b+\underset{g,i,j}{\sum }\frac{U_{g,i,j}^{2}}{2}\right)\tau \right)\\ \;\;\;\;\sim Gamma\left(a+\frac{J\underset{g}{\sum }I_{g}}{2},b+\underset{g,i,j}{\sum }\frac{U_{g,i,j}^{2}}{2}\right). \end{array}$$

We pool all the samples from *U_g,i,j _*and get its estimate U^g,i,j=1T-tb+1 ∑tbTUg,i,jt, where $U_{g,i,j}^{t}$ denotes sampled *U_g,i,j _*at sample *t. t_b _*is the last sample when burn-in stops. *T *is the total number of Gibbs samples. U^g,i,j will be passed to the Gamma-Gamma model to estimate parameters associated with DEG identification.

Similarly for the Gamma-Gamma model, we sample the parameters **β, λ**, α, α_0_, *v *and **d **according to their conditionals. The posterior distribution of $\beta_{j}\left(\beta_{j_{1}},\beta_{j_{2}}\right)$ can be sampled from:

(14) P(βg,j1|yg,j1,λg(1),α)~Gamma∑iyg,i,j1+α, ∑ixg,iexpU^g,i,j1+λg(1),

(15) P(βg,j2|yg,j2,λg(2),α)~Gamma∑iyg,i,j2+α, ∑ixg,iexpU^g,i,j2+λg(2).

If *d_g _*= 1,

(16) $$\text{P}\left(\lambda_{g}^{\left(1\right)}|\beta_{g}^{\left(1\right)},\nu ,\alpha ,\alpha_{0}\right)\sim Gamma\left(J_{1}\alpha +\alpha_{0},\underset{j_{1}}{\sum }\beta_{g,j_{1}}+\nu \right),$$

(17) $$\text{P}\left(\lambda_{g}^{\left(2\right)}|\beta_{g}^{\left(2\right)},\nu ,\alpha ,\alpha_{0}\right)\sim Gamma\left(J_{2}\alpha +\alpha_{0},\underset{j_{2}}{\sum }\beta_{g,j_{2}}+\nu \right);$$

If *d_g _*= 0,

(18) $$\text{P}\left(\lambda_{g}^{\left(1\right)}|\beta_{g},\nu ,\alpha ,\alpha_{0}\right)=P\left(\lambda_{g}^{\left(2\right)}|\beta_{g},\nu ,\alpha ,\alpha_{0}\right)\sim Gamma\left(J\alpha +\alpha_{0},\underset{j}{\sum }\beta_{g,j}+\nu \right).$$

The posterior distribution of ν follows Gamma distribution that is given by:

(19) $$\text{P}\left(\nu |\lambda ,\text{d},\alpha ,\alpha_{0}\right)\sim Gamma\left(\left(G+\underset{g}{\sum }d_{g}\right)\alpha_{0}+a_{0},\underset{g}{\sum }\left(\lambda_{g}^{\left(1\right)}+\lambda_{g}^{\left(2\right)}\times d_{g}\right)+b_{0}\right).$$

According to Wei *etal*. [14], the posterior distribution of **d **given **β**, *α, α*_0 _and ν can be derived as:

(20) $$\begin{array}{c} P\left(d_{g}|\beta ,\alpha ,\alpha_{0},\nu \right)={\left(K_{1}K_{2}\frac{{\left(\underset{j_{1}}{\prod }\beta_{g,j_{1}}\times \underset{j_{2}}{\prod }\beta_{g,j_{2}}\right)}^{\alpha -1}}{{\left(\nu +\underset{j_{1}}{\sum }\beta_{g,j_{1}}\right)}^{J_{1}\alpha +\alpha_{0}}{\left(\nu +\underset{j_{2}}{\sum }\beta_{g,j_{2}}\right)}^{J_{2}\alpha +\alpha_{0}}}\right)}^{d_{g}}\\ \;\;\;\;\;\;\;\times {\left(K\frac{{\left(\underset{j_{1}}{\prod }\beta_{g,j_{1}}\times \underset{j_{2}}{\prod }\beta_{g,j_{2}}\right)}^{\alpha -1}}{{\left(\nu +\underset{j_{1}}{\sum }\beta_{g,j_{1}}+\underset{j_{2}}{\sum }\beta_{g,j_{2}}\right)}^{\left(J_{1}+J_{2}\right)\alpha +\alpha_{0}}}\right)}^{1-d_{g}}\times \pi_{g}, \end{array}$$

where

$$K_{1}=\frac{\nu^{\alpha_{0}}\text{Γ}\left(J_{1}\alpha +\alpha_{0}\right)}{{\text{Γ}}^{J_{1}}\left(\alpha \right)\text{Γ}\left(\alpha_{0}\right)},K_{2}=\frac{\nu^{\alpha_{0}}\text{Γ}\left(J_{2}\alpha +\alpha_{0}\right)}{{\text{Γ}}^{J_{2}}\left(\alpha \right)\text{Γ}\left(\alpha_{0}\right)},\text{and }K=\frac{\nu^{\alpha_{0}}\text{Γ}\left(\left(J_{1}+J_{2}\right)\alpha +\alpha_{0}\right)}{{\text{Γ}}^{J_{1}+J_{2}}\left(\alpha \right)\text{Γ}\left(\alpha_{0}\right)}.$$

The posterior distribution of *α*_0 _and *α *are given by:

(21) $$P\left(\alpha_{0}|\nu ,\lambda ,\text{d}\right)\sim \underset{g}{\prod }\left(\frac{\nu^{\alpha_{0}}}{\text{Γ}\left(\alpha_{0}\right)}{\left(\lambda_{g}^{\left(1\right)}\right)}^{\alpha_{0}-1}\times {\left(\frac{\nu^{\alpha_{0}}}{\text{Γ}\left(\alpha_{0}\right)}{\left(\lambda_{g}^{\left(2\right)}\right)}^{\alpha_{0}-1}\right)}^{d_{g}}\right),$$

(22) $$P\left(\alpha |\beta ,\lambda \right)\sim \underset{g}{\prod }\left(\underset{j_{1}}{\prod }\frac{\left(\lambda_{g}^{\left(1\right)}\right)}{\text{Γ}\left(\alpha \right)}\beta_{g,j_{1}}^{\alpha -1}\times \underset{j_{2}}{\prod }\frac{{\left(\lambda_{g}^{\left(2\right)}\right)}^{\alpha }}{\text{Γ}\left(\alpha \right)}\beta_{g,j_{2}}^{\alpha -1}\right).$$

We use Gibbs sampling method [4,13,15,16] to estimate the posterior distributions of individual parameters iteratively from their complex joint distribution. For parameters **β**, *ν, τ*, and **λ**, we use conjugate priors to sample from their conditional distributions with standard probability distributions (Gamma distribution). For parameters (**U**, *α*_0 _and *α*) that do not have conjugate priors, we use Metropolis-Hastings sampling to sample their posterior distributions. Please see more details about the implementation of Gibbs sampler in additional file 1.

## Results and discussion

### Over-dispersion of RNA-Seq counts in two dimensions: between-sample variation and within-sample variation

**Figure 2:**
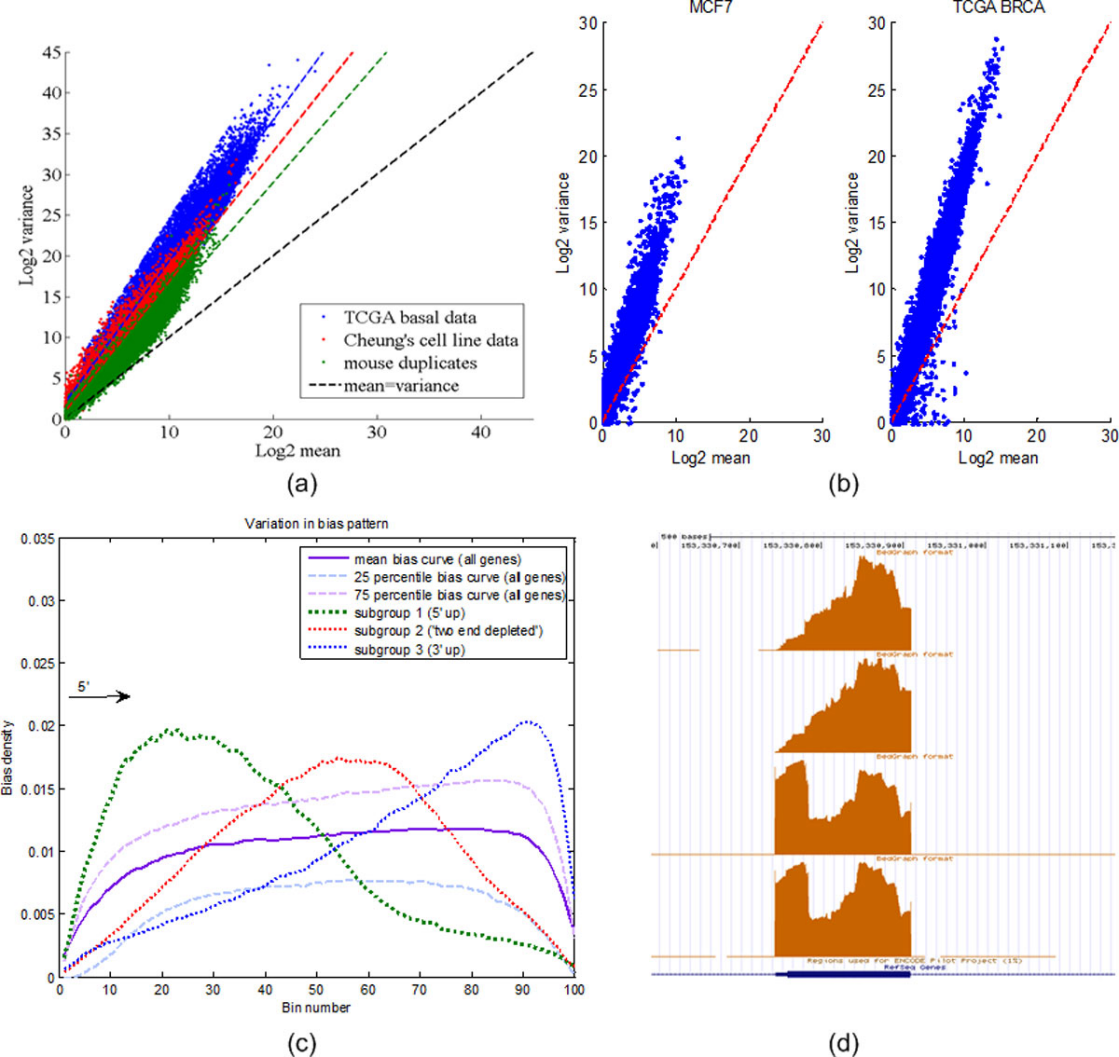
**Between-sample variation and within-sample variation in RNA-Seq data**.(a) Three datasets are used to show the over-dispersion of reads across samples. Scatter plots are in log 2 scale. (b) In both MCF7 breast cancer samples and TCGA breast cancer patient samples, we observe that the variance of read counts is significantly larger than the average counts in 100nt bins. (c) Bias patterns of genes in the same sample are further dissected. In contrast to a general right-tailed (biased towards 3'-end of transcript) coverage, we also observe groups of genes that have either biased expression towards the 5'-end, or depleted expression in both ends. (d) An example to show variation of sequencing bias among biological samples.

Even though RNA-Seq has been proved to be more accurate and less sensitive to background noise than traditional microarray technology [17], large variance in sequencing counts has complicated the detection of hidden biological signals. Increasing evidence shows that read counts in RNA-Seq data have much larger variance than the mean (i.e., 'over-dispersion'), which requires replacing the Poisson model with more sophisticated count models such as Negative Binomial model [8]. Figure 2 (a) shows the scatter plot of mean versus variance for three RNA-Seq datasets: basal breast cancer samples from The Cancer Genome Atlas (TCGA) project [18], human B cell datasets from Cheung et al.[19], and a mouse dataset [20]. The slopes of the least squares (LS) fit lines for all scatter plots are apparently larger than the Poisson model, which implies severe over-dispersion in all three RNA-seq datasets. In addition to variability across samples ('between-sample variation'), we have also observed strong variance of sequencing counts among genomic loci in the same biological sample ('within-sample variability'). Figure 2 (b) shows the scatter plots of counts that fall in 100nt bins along the same gene within the same sample. One TCGA breast cancer sample and one MCF7 breast cancer cell line sample [21] are used as examples. Figure 2 (b) shows strong within-sample over-dispersion of read counts in both RNA-Seq samples, implying the presence of unknown sources of variability. Figure 2 (c) shows the variation of sequencing bias among all the genes within one sample. Despite an overall tendency where read coverage is biased towards the 3'-end of the transcript, subgroups of the genes in the genome exhibit diverse patterns showing either a bias towards the 5'-end or having depleted coverage on both ends. In Figure 2 (d), we further show an example of read coverage for gene S100A9 (exon 2) across four samples from TCGA basal breast cancer dataset. The base level coverage has two distinct patterns, indicating large variation of unknown read bias in the same group. The ambiguity in coverage pattern cannot be explained by deterministic systematic bias, and therefore need to be corrected for accurate estimation of RNA-Seq abundance.

### Generate simulation data based on real RNA-Seq datasets

**Figure 3:**
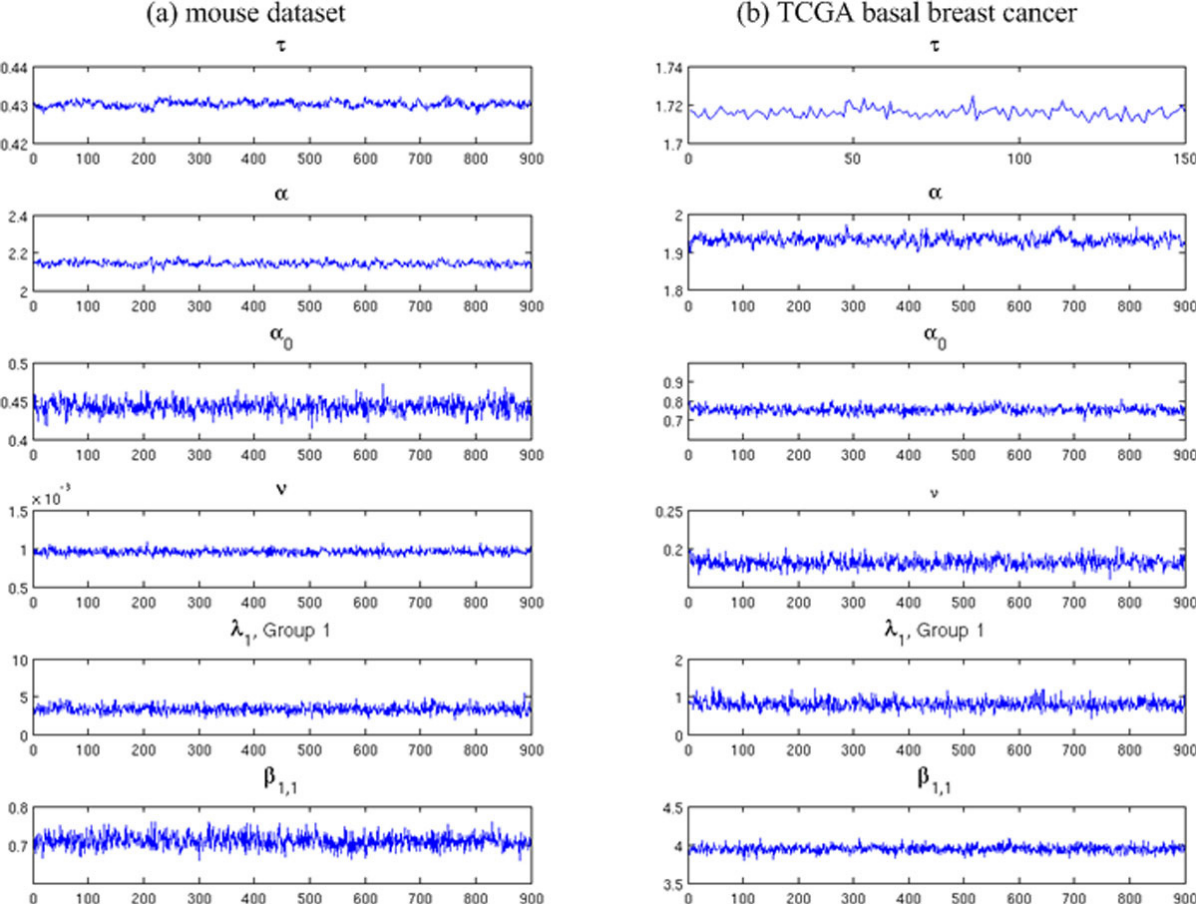
**Estimate of model parameters from real datasets**.(a) Model parameters estimated from mouse dataset. (b) Model parameters estimated from TCGA basal breast cancer dataset. For most parameters, we record 900 samples (thin = 10, i.e., record every 10^th ^sample) after the first 100 burn-in samples. An exception was made for learning parameter *τ *in TCGA dataset to reduce memory usage in estimating Poisson-Lognormal regression model (200 samples were recorded, among which first 50 were discarded as burn-in).

To generate realistic synthetic data that represent characteristics of real RNA-Seq data, we adopted a simulation strategy proposed by Wu *et al*. [10] to first estimate model parameters from real datasets and then use them to generate sequencing counts based on human annotation file (version: GRCh37/hg19). Two RNA-Seq datasets were used in the study: 1) a mouse dataset with 10 C57BL/6J (B6) mouse samples and 11 DBA/2J (D2) mouse samples [20]; 2) 23 basal breast cancer samples from the TCGA project [18]. For the TCGA dataset, we divided the patients (which received chemotherapy treatment) into two groups: early re-current group (recurrence time < 2 yrs, 13 samples) and late recurrent group (recurrence time > 3 yrs, 10 samples). Figure 3 gives the trace plots of sampled model parameters. We used thin = 10 for the Gibbs sampling process, which means to record every 10^th ^sample. It took BADGE several hours to estimate posterior distributions using 10,000 iterations on real dataset. We have also plotted auto-correlation curves of each parameter in supplementary materials (additional file 1). We further explored the variability of RNA-Seq data by close examination of estimated model parameters. For within-sample variability, two typical values of over-dispersion prior parameter *τ *have been estimated, where *τ *= 0.44 in mouse dataset and *τ *= 1.78 in TCGA dataset. In our Poisson-Lognormal regression model, *τ *is the precision parameter (inverse of Gaussian variance *σ*^2^) that controls overall degree of within-sample over-dispersion. Smaller *τ *indicates larger variation of read counts across the transcriptome. Small value of *τ *observed in real datasets strongly supported our motivation that read counts along one transcript must be corrected for improved abundance estimation. Between-sample variability is jointly determined by *α, α*_0_, and *ν*. Small value in *ν *(0.001 in mouse and 0.2 in TCGA sample) yield large variation of VAR (*λ*), where VAR (*β*) increases when *λ *takes small value. We used the model parameters estimated from real datasets to generate read counts for simulation. Please refer to supplementary materials in additional file 1 for more detailed description of simulation data.

### Performance comparison for abundance estimation on simulation data

**Figure 4:**
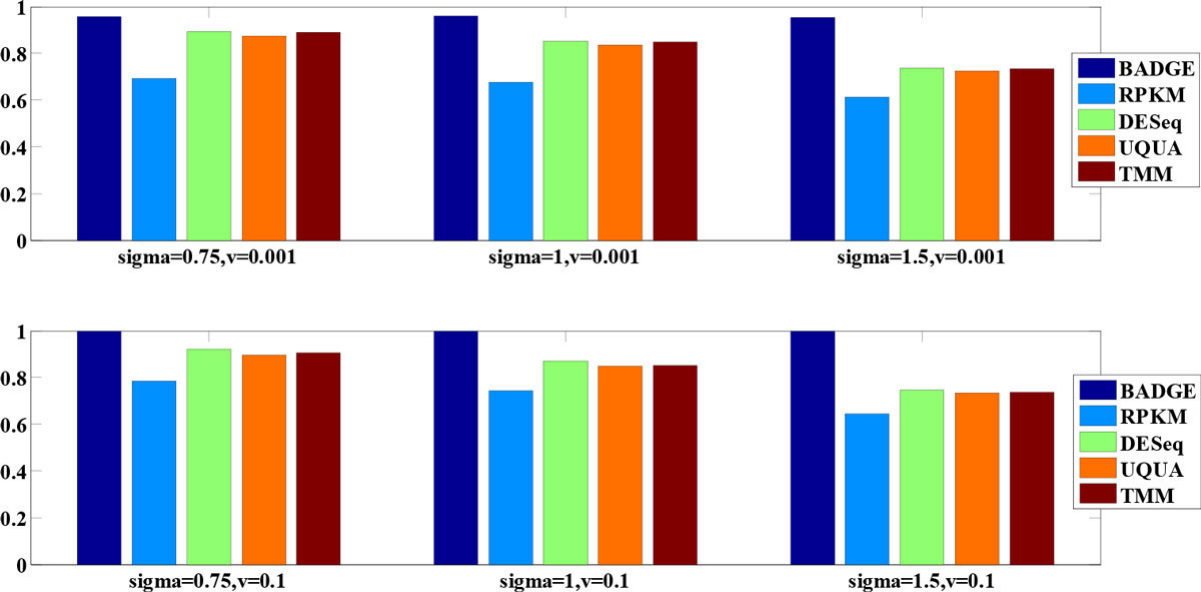
**Performance comparison for abundance estimation on simulation data**.

We incorporated the estimated parameter *τ *from real datasets to generate synthetic data and studied the performance of the BADGE method for abundance estimation. We compared our method with four commonly used methods for RNA-Seq normalization, which were: reads-per-kilobase-per-million (RPKM), DESeq, trimmed mean of M-values (TMM) and upper quantile normalization (UQUA). RPKM [22] is calculated through normalizing reads by length of genomic features (genes and exons) and total library size. DESeq normalization is implemented by DESeq (1.14.1) [9], which is a differential gene identification method based on negative binomial model. TMM was originally developed in edgeR [8] and later included into BioConductor package NOISeq [23]. NOISeq (2.0.0) also has separate implementations of RPKM and UQUA methods, which were used here for performance comparison. Based on estimated parameters from real RNA-Seq datasets (mouse and TCGA breast cancer data), we selected typical model parameters to simulate count data: σ = 0.75, 1 and 1.5; ν = 0.1 and 0.001; α = 2 and α_0 _= 0.5. We used the average correlation of normalized counts with ground truth gene expression to measure the accuracy of abundance quantification across multiple samples. Figure 4 gives the average correlation for all competing methods under different model settings. From Figure 4, we see that the BADGE method had robust performance under different over-dispersion settings by maintaining a performance measure (correlation to ground truth) very close to 1. Among the rest of the normalization methods, DESeq, TMM and UQUA achieved comparable performance across multiple parameter settings, while RPKM had the least favourable performance in all scenarios. Our computational result is quite consistent with the observation by Dillies *et al*. [24] that DESeq and TMM (edgeR) are much better normalization methods than RPKM.

### Performance comparison for differentially expressed gene (DEG) identification on simulation data

We compared BADGE with four existing methods: DESeq (1.14.1, fitType=local), edgeR (3.4.2, default), DSS (2.0.0, default), EBSeq (1.3.1, default), for differentially expressed gene (DEG) identification from RNA-Seq data. We used parameters learned from real datasets to generate simulation data. Within-sample variability is controlled by precision parameter *τ *(or standard deviation σ), and we set *τ *= 1.78 (i.e., *σ *= 0.75, which was learned from TCGA basal dataset) and *τ *= 0.44 (i.e., *σ *= 1.5, which was learned from mouse dataset.). According to estimated parameters from real datasets, we set *α *= 2, *α*_0 _= 0.5, while varied ν between 0.001 and 0.1, which was consistent with the parameter settings in abundance estimation. For each parameter set, we randomly selected 10 genesets from hg19 annotation file to evaluate the variance of the performance. Area-under-the-curve (AUC) of the receiver operating characteristic (ROC) curve was used as performance measurement. Tables 1, 2, 3 give the AUC values for each method along with standard deviations (listed in parentheses) of AUCs across 10 experiments.

**Table 1:** Performance comparison using AUC for DEG identification (highly differentially expressed genes)

σ (τ)	*ν*	BADGE	DESeq	edgeR	DSS	EBSeq
0.75	0.001	**0.939 **	0.921	0.919	0.881	0.928
(1.78)		(0.018)	(0.017)	(0.018)	(0.055)	(0.017)
	0.1	**0.918 **	0.894	0.895	0.889	0.906
		(0.018)	(0.018)	(0.020)	(0.024)	(0.021)
1.5	0.001	**0.925 **	0.881	0.875	0.809	0.890
(0.44)		(0.014)	(0.029)	(0.032)	(0.082)	(0.030)
	0.1	**0.924 **	0.868	0.865	0.858	0.875
		(0.023)	(0.027)	(0.025)	(0.027)	(0.022)

**Table 2:** Performance comparison using AUC for DEG identification (moderately differentially expressed genes)

σ (τ)	*ν*	BADGE	DESeq	edgeR	DSS	EBSeq
0.75	0.001	**0.901**	0.724	0.753	0.739	0.750
(1.78)		(0.071)	(0.070)	(0.081)	(0.197)	(0.069)
	0.1	**0.905 **	0.768	0.760	0.852	0.781
		(0.076)	(0.051)	(0.066)	(0.071)	(0.044)
1.5	0.001	**0.882 **	0.691	0.699	0.790	0.725
(0.44)		(0.056)	(0.041)	(0.042)	(0.088)	(0.035)
	0.1	**0.890 **	0.743	0.729	0.798	0.748
		(0.080)	(0.070)	(0.081)	(0.074)	(0.058)

**Table 3:** Performance comparison using AUC for DEG identification (weakly differentially expressed genes)

σ (τ)	*ν*	BADGE	DESeq	edgeR	DSS	EBSeq
0.75	0.001	**0.793 **	0.656	0.680	0.603	0.687
(1.78)		(0.099)	(0.058)	(0.084)	(0.133)	(0.031)
	0.1	**0.778 **	0.659	0.663	0.695	0.684
		(0.092)	(0.043)	(0.062)	(0.075)	(0.049)
1.5	0.001	**0.806 **	0.641	0.647	0.635	0.669
(0.44)		(0.080)	(0.028)	(0.071)	(0.134)	(0.029)
	0.1	**0.823 **	0.687	0.680	0.724	0.682
		(0.109)	(0.072)	(0.063)	(0.104)	(0.073)

We simulated RNA-Seq gene expression with three different scenarios: genes that were highly differentially expressed, moderately differentially expressed and weakly differentially expressed (see supplementary materials in additional file 1 for more information). From Tables 1, 2, 3, we see that the BADGE method consistently outperformed existing methods in different parameter settings (highlighted in bold). For highly differentially expressed genes (Table 1), the performance of the other methods degraded as we decreased *τ*, while BADGE was able to maintain good performance by employing a Poisson-Lognormal model to account for within-sample variability. For genes that were weakly differentially expressed (Table 3), BADGE achieved a maximum improvement of AUC up to 1.3, compared to the second best method EBSeq (Table 3, *τ *= 0.44, *ν *= 0.001).

### Performance comparison for differentially expressed gene (DEG) identification on Sequencing Quality Control (SEQC) data

**Figure 5:**
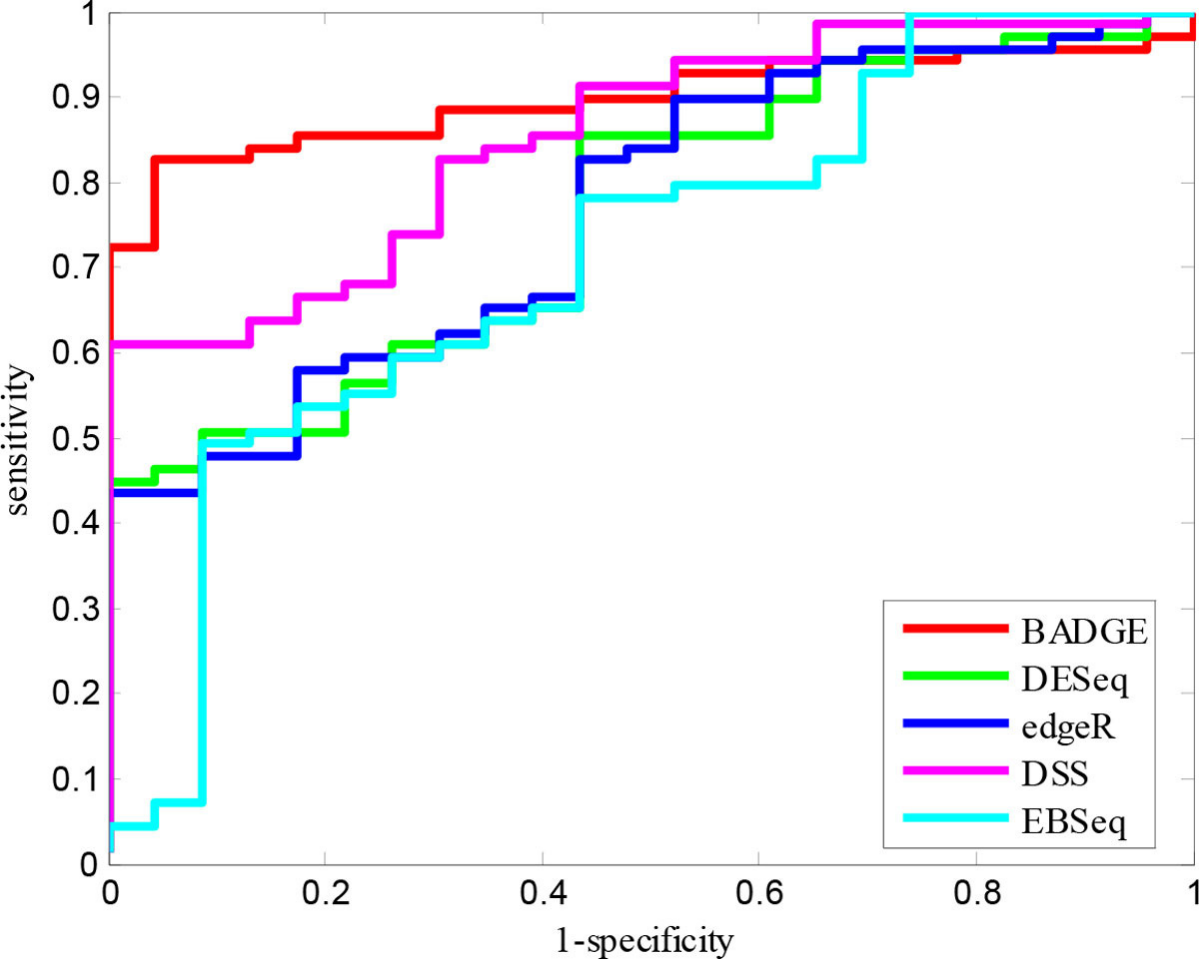
**Performance comparison for differentially expressed gene identification on the SEQC dataset**.

We compared the performance of BADGE with existing RNA-Seq differential gene identification methods on the Sequencing Quality Control (SEQC) dataset with ERCC spike-in controls [25]. 92 artificial transcripts were mixed into a real RNA-Seq library with different ratios (1:1 for none differentially expressed genes, and 4:1, 2:3 and 1:2 for differentially expressed genes), which were used as ground truth differential states for differential gene identification. Gene level counts were downloaded from http://bitbucket.org/soccin/seqc. We compared the ROC curves between BADGE and four other methods used in the simulation study for differential gene identification, which were DESeq [9], edgeR [8], DSS [10] and EBSeq [11]. Figure 5 shows the ROC curves of the five competing methods.

On the SEQC dataset, BADGE had the best performance among all five methods by achieving an AUC very close to 0.9. The second best method was DSS with an AUC about 0.85. DESeq (AUC = 0.7624) and edgeR (AUC = 0.7675) had very close performance, which was consistent with the previous results reported by Rapaport *et al*. [25]. EBSeq, on the other hand, had the least favourable performance (AUC = 0.71) on this specific dataset and it failed to detect the most strongly differentially expressed genes: its sensitivity was less than 0.1 when its specificity was about 0.9. By close examination of the 'left' ROC curves of the five methods, we can further infer that BADGE should have significantly better precision than the competing methods, whose sensitivity went all the way up to 0.7 before any sacrifice in specificity.

### 'Blind' estimation of hidden heterogeneity in RNA-Seq data

**Figure 6:**
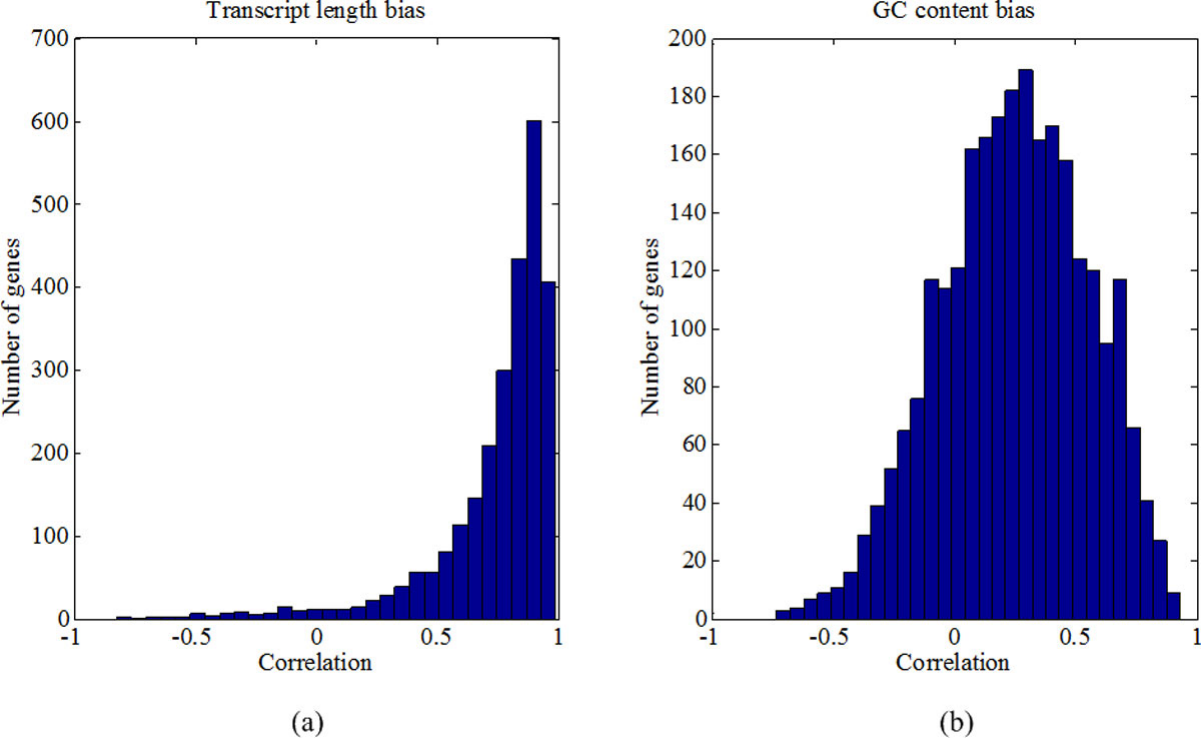
**Dissecting read variability into sequencing bias**.(a) Estimated over-dispersion parameter *U*_*g,i,j *_shows strong correlation with transcript location. (b) GC content bias can also be inferred from *U*_*g,i,j*_. Top expressed 2631 genes (number-of-exons>10) from one TCGA basal sample are used in this study.

In contrast to uniform random sampling, read counts in RNA-Seq data show large variance due to different sources of hidden heterogeneities. By using a Poisson-Lognormal regression model, BADGE can 'blindly' estimate hidden heterogeneities across the transcriptome to minimize overall variability in sequencing counts without using additional information (e.g., genome sequence information in huge Fasta file to calculate GC percentage). In BADGE model, the variability of each individual exon is carried by parameter *U*_*g,i,j *_(gene *g*, exon *i*, sample *j*), while the overall degree of variation is controlled by *τ*. Based on sampled parameters from real datasets, we further investigated *U*_*g,i,j *_to see how systematic artifacts in RNA-Seq (such as transcript length bias and GC content) can be de-convoluted by BAGDE method. Figure 6 shows the histogram of Pearson's correlation between estimated *U*_*g,i,j *_and: 1. transcript location; 2. GC content. From Figure 6 (a), we see strong positive correlation between estimated over-dispersion parameter *U_g,i,j_* and transcript location, which indicates that most of the genes had biased expression towards 3'-end of the transcript in our dataset, while about 15 percent of the genes had low correlation (<0.5). In addition, a significant correlation between *U*_*g,i,j *_and GC-content bias (extremely low or high GC content are associated with low abundance [26]) were also observed in Figure 6 (b). These observations support that BADGE can correctly estimate hidden sources of variation in RNA-Seq data 'blindly' without using transcript location information or sequence information.

## Conclusions

Large variation in RNA-Seq data has become the major obstacle against accurate estimation of gene expression and DEG identification. Much effort has been made to model variation across biological replicates, while limited attention is paid to tackle extensive over-dispersion observed in sequencing counts. For short-read sequencing technologies (e.g., Illumina), multiple sources of systematic bias have been identified, including transcript length bias, GC-content bias, etc. However, in-depth investigation of real RNA-Seq datasets has revealed the following complications: 1) Sequencing bias not only changes from one gene to another, but also varies among samples (Figure 2(c) and (d)); 2) Gene expression is jointly influenced by multiple bias factors, which leads to large variation across the entire transcriptome (Figure 6). However, current research activities have been focused on addressing individual bias corrections, which lacks a unified effort to account for total variability in RNA-Seq data. Therefore, we propose the BADGE method to extensively model both within-sample variability (bias and random variance) and between-sample variability (biological variations among replicates or within the same phenotype group) to improve quality of inference.

## Competing interests

The authors declare that they have no competing interests.

## Declarations

The publication costs for this article were funded by National Institutes of Health (NIH) [CA149653].

## Authors' contributions

JG and JX designed and implemented the algorithm. JG, XW, JX designed and performed the computational experiments. JG and JX contributed to the writing. LHC and RC provided their biological guidance on the breast cancer study. All authors read and approved the final manuscript.

## Supplementary Material

Additional file 1 **Supplementary materials**. This additional file contains supplementary information for the main text, including parameter setting for BADGE method, computational implementation, simulation design, supplementary figures and tables, etc. Click here for file
