# Supplementary material for: BADGE: A novel Bayesian model for accurate abundance quantification and differential analysis of RNA-Seq data
Source: BMC Bioinformatics. 2014 Sep 10;15(Suppl 9):S6. doi: 10.1186/1471-2105-15-S9-S6 (PMC4168709; doi:10.1186/1471-2105-15-S9-S6)
Supplement: Additional file 1 — Supplementary materials. This additional file contains supplementary information for the main text, including parameter setting for BADGE method, computational implementation, simulation design, supplementary figures and tables, etc. [file 1471-2105-15-S9-S6-S1.pdf]

Supplementary Materials

BADGE: A novel Bayesian model for accurate abundance quantification and differential analysis of RNA-Seq data

Jinghua Gu[1], Xiao Wang[1], Leena Halakivi-Clarke[2], Robert Clarke[2], Jianhua Xuan[1§]

[1] Department of Electrical and Computer Engineering, Virginia Polytechnic Institute and State University, Virginia, USA

[2] Department of Oncology, Lombardi Comprehensive Cancer Center, Georgetown University, Washington, DC, USA

[§]Corresponding author

Email addresses:

JG: gujh@vt.edu

XW: wangxiao@vt.edu

LHC: clarkel@georgetown.edu

RC: clarker@georgetown.edu

JX: xuan@vt.edu

Supplementary Methods & Results

Implementation of Gibbs sampling for BADGE

We use Gibbs sampling to draw samples of the model parameters from their posterior distributions. For parameters $\boldsymbol{\beta}$, v, τ, and $\boldsymbol{\lambda}$, we use conjugate priors to sample from their conditional distributions with standard probability distributions (Gamma distribution). For parameters $\mathbf{U}$, α_0, and α, where no conjugate priors can be used, Metropolis-Hastings (MH) sampling with random walk proposal function is used to draw samples from their conditionals. However, selection of proposal function is critical to the efficiency of MH sampling. We start from Normal N(0,1) distribution for all parameters in MH sampling and 'adaptively' learn proper proposal standard deviation by tuning for optimal acceptance rate. According to Roberts and Rosenthal [1], an acceptance rate of 0.15 to 0.5 may yield a sampling efficiency of 80%. The MH sampling process consists of two stages: an 'adaptive stage' to learn proposal scale and a 'stable stage' to estimate posterior distributions. For Bernoulli parameter **d**, state '0' and '1' are selected based on their conditional distributions.

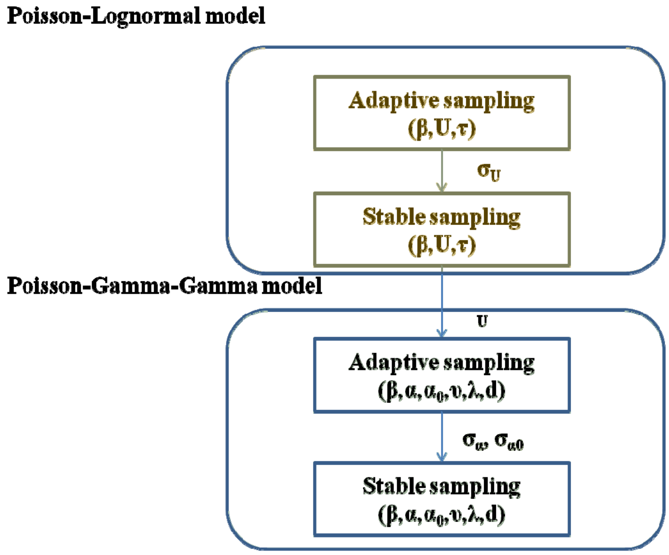

Figure S1. Implementation of Gibbs sampler for BADGE. σ_U, σ_α, and $\sigma_{\alpha 0}$ are the standard deviation of proposal functions for sampling U, α, and α_0, respectively.

We have made practical adjustments for the implementation of BADGE model so that the method can enjoy improved efficiency without compromise of performance. The computational burden of the BADGE method mainly comes from Poisson-Lognormal regression model, where each exon is associated with one parameter $U_{g,i,j}$ to model within-sample variability. In this case, for 10,000 genes each with 10 exons in 10 samples, we will need to sample 1000,000 $U_{g,i,j}$ in one iteration of Gibbs sampling. Upon further investigation of the joint computational model, we see that $U_{g,i,j}$ has relatively small range along the positive axis near 0 due to the exponential term in Equation (1). This makes the sampling process of $U_{g,i,j}$ theoretically easier. It has been confirmed by both simulation and real data analysis that $U_{g,i,j}$ typically converges well in hundreds of iterations. On the other hand, parameters in Gamma-Gamma model do not necessarily have this nice property so that we usually need more Gibbs samples to guarantee the convergence (especially for differential state **d**). To accommodate the convergence property of different parameters, we 'split' the BADGE model into two sub-models, where within-sample over-dispersion parameters are estimated first (through hundreds of iterations) and then fixed for differential analysis (10,000 iterations). Simulation result shows that the implementation of the two sub-models will not affect the performance of the algorithm in general (in Figure 4 of the main text, performance of BADGE remains robust for different levels of within-sample variability), while it can reduce overall running time. Therefore, we can practically reduce computational cost of BADGE on larger real datasets by sacrificing some integrity of the original Bayesian model. Figure S1 gives a simple block diagram of how Gibbs sampling is implemented.

Figure S1 shows that we first implement the Poisson-Lognormal regression sub-model to estimate $U_{g,i,j}$, which is later passed to the Poisson-Gamma-Gamma model for differential gene analysis. In each sub-model, a two-stage (adaptive stage and stable stage) MH sampling is implemented for parameters with no conjugate priors. In the adaptive stage, we start from $N\left(1,\sigma^2\right)$ with $\sigma = 1$, and fine-tune σ for every 100 iterations based on the acceptance rate. The target acceptance rate is set as 0.4 [1, 2] and we use the following scheme to update the proposal scale σ [1]:

$$\sigma_{new} = \frac{\sigma_{cur}\Phi^{-1}\left(p_{\text{opt}}/2\right)}{\Phi^{-1}\left(p_{cur}/2\right)}, \tag{S1}$$

where σ_{cur} and σ_{new} is the current and new proposal scale. p_{opt} is the target acceptance rate, which is 0.4 in our case. p_{cur} is the current acceptance rate. Φ^{-1} is the normal inverse cumulative distribution. After several (e.g., 5 stages, each with 100 iterations) iterations of adjustment, we fix the proposal scale σ and use it to re-sample the sub-model until convergence (hundreds iterations for Poisson-Lognormal regression model and thousands of iterations for Poisson-Gamma-Gamma model).

Design of simulation study

As we have discussed in the main text, we used the model parameters estimated from real datasets to generate read counts in our simulation. For abundance estimation, we varied model parameter v to generate simulation data with different levels of between-sample over-dispersion. Within-sample over-dispersion parameter τ (or σ) was also adjusted across a wide dynamic range to cover a complete spectrum of RNA-Seq variability. Human RefSeq annotation file was used in the simulation and we generated a simulation dataset including more than 200 genes and 20 samples for each

parameter set (α and τ). Finally, we calculated the mean of correlation of estimated gene abundance with ground truth value to report in Figure 4.

For the simulation comparing differentially expressed gene (DEG) identification, we simulated count datasets with different levels of differentially expressed genes. This detailed 'breakdown' of simulation data was very helpful for us to investigate the impact of count variation in RNA-Seq data on DEG identification. The degree of differential expression of one gene between condition 1 and 2 was controlled by model parameter $\boldsymbol{\lambda}^{(1)}$ and $\boldsymbol{\lambda}^{(2)}$. We simulated $\boldsymbol{\lambda}^{(1)}$ and $\boldsymbol{\lambda}^{(2)}$ with different correlation levels, where low (high) correlation of $\boldsymbol{\lambda}^{(1)}$ and $\boldsymbol{\lambda}^{(2)}$ corresponds to strong (weak) differential expression. In order to control correlation of $\boldsymbol{\lambda}^{(1)}$ and $\boldsymbol{\lambda}^{(2)}$ at an exact level, we first sorted both vectors $\boldsymbol{\lambda}^{(1)}$ and $\boldsymbol{\lambda}^{(2)}$ generated from original BADGE model in descending order. In this case, the rank correlation of $\boldsymbol{\lambda}^{(1)}_{sort}$ and $\boldsymbol{\lambda}^{(2)}_{sort}$ became 1 and the expression difference of any gene among two groups was minimized. Next, we fixed $\boldsymbol{\lambda}^{(1)}_{sort}$ and circularly shifted values in $\boldsymbol{\lambda}^{(2)}_{sort}$ by K genes to get $\boldsymbol{\lambda}^{(2)}_{shiftK}$, where the correlation between $\boldsymbol{\lambda}^{(1)}_{sort}$ and $\boldsymbol{\lambda}^{(2)}_{shiftK}$ decreased accordingly. Through the above procedure, we could flexibly control the degree of differential expression by either increase or decrease the correlation between $\boldsymbol{\lambda}^{(1)}_{sort}$ and $\boldsymbol{\lambda}^{(2)}_{shiftK}$ without changing the distributions of model parameters ($\boldsymbol{\lambda}^{(1)}_{sort}$ and $\boldsymbol{\lambda}^{(2)}_{shiftK}$ are i.i.d. distributions of $\boldsymbol{\lambda}^{(1)}$ and $\boldsymbol{\lambda}^{(2)}$). In our simulation study, the correlation levels of $\boldsymbol{\lambda}^{(1)}$ and $\boldsymbol{\lambda}^{(2)}$ were: -0.025 (strongly differentially expressed), 0.364 (moderately differentially expressed), and 0.556 (weakly differentially expressed).

Supplementary Figures

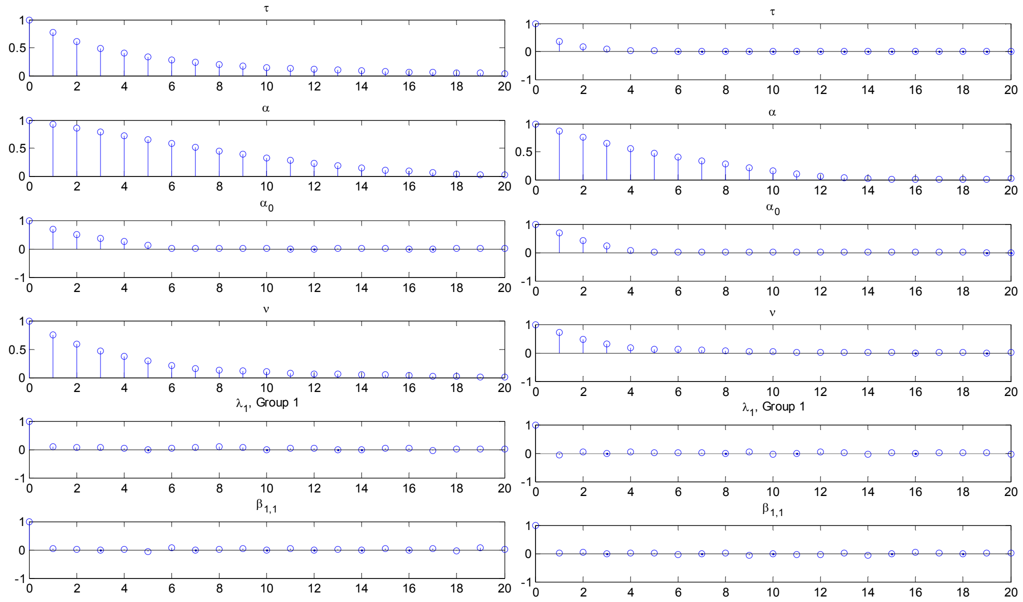

(a) mouse data (b) TCGA basal breast cancer

Figure S2. Autocorrelation plots for learned model parameters in real datasets. The autocorrelation of model parameters estimated from the Gibbs sampler typically drops to zero in shift of 10 samples. Particularly, for parameters that are sampled from conjugate prior distributions (e.g., $\boldsymbol{\lambda}$ and $\boldsymbol{\beta}$), the dependency between consecutive samples is very low (autocorrelation drops to 0 after 1 sample shift), showing a high efficiency of the sampling process.

Supplementary Tables

Table S1 Model parameters and hyper-parameter selection

model parameter	hyper-parameter	prior distribution	Description
d_g	π_g	$P(d_g=1)=\pi_g=0.5$<br>$P(d_g=0)=1-\pi_g=0.5$	$p(d_g)$ follows Bernoulli distribution with equal probability of taking 0 and 1.
ν	a_0, b_0	$\nu \sim \text{Gamma}(a_0, b_0)$<br>$a_0=1$, $b_0=0$	$p(\nu)$ has 'flat' Gamma prior on the positive real axis*
α_0	N/A	$\alpha_0 \sim I(0,\infty)$**	Non-informative flat prior
α	N/A	$\alpha \sim I(0,\infty)$	Non-informative flat prior
τ	a, b	$\tau \sim \text{Gamma}(a, b)$<br>$a=1$, $b=0$	$p(\tau)$ has 'flat' Gamma prior on the positive real axis*

*: we assume Gamma 'flat' prior for parameter ν and τ for computational convenience so that their posterior distributions will also follow Gamma distribution, which is a known distribution that can be easily sampled in practice.
**: $I(0,\infty)$ denotes the non-informative prior defined over entire positive real axis [3].

References

1. Roberts GO, Rosenthal JS: **Optimal Scaling for Various Metropolis-Hastings Algorithms**. *Statistical Science* 2001, **16**(4):17.
2. **SAS/STAT(R) 9.2 User's Guide, Second Edition** [http://support.sas.com/documentation/cdl/en/statug/63033/HTML/default/viewer.htm#statug_mcmc_sect022.htm]
3. Hu M, Zhu Y, Taylor JM, Liu JS, Qin ZS: **Using Poisson mixed-effects model to quantify transcript-level gene expression in RNA-Seq**. *Bioinformatics* 2012, **28**(1):63-68.
